# Fruit cuticular waxes as a source of biologically active triterpenoids

**DOI:** 10.1007/s11101-012-9241-9

**Published:** 2012-06-26

**Authors:** Anna Szakiel, Cezary Pączkowski, Flora Pensec, Christophe Bertsch

**Affiliations:** 1 Department of Plant Biochemistry, Faculty of Biology, University of Warsaw, ul. Miecznikowa 1, 02-096 Warszawa, Poland; 2 UFR Pluridisciplinaire Enseignement Professionnalisant Supérieur, Laboratoire Vigne Biotechnologie et Environnement EA 3391, Université de Haute-Alsace, 33, rue de Herrlisheim, 68000 Colmar, France

**Keywords:** Cuticular waxes, Fruit peel, Health benefits, Triterpenoids

## Abstract

The health benefits associated with a diet rich in fruit and vegetables include reduction of the risk of chronic diseases such as cardiovascular disease, diabetes and cancer, that are becoming prevalent in the aging human population. Triterpenoids, polycyclic compounds derived from the linear hydrocarbon squalene, are widely distributed in edible and medicinal plants and are an integral part of the human diet. As an important group of phytochemicals that exert numerous biological effects and display various pharmacological activities, triterpenoids are being evaluated for use in new functional foods, drugs, cosmetics and healthcare products. Screening plant material in the search for triterpenoid-rich plant tissues has identified fruit peel and especially fruit cuticular waxes as promising and highly available sources. The chemical composition, abundance and biological activities of triterpenoids occurring in cuticular waxes of some economically important fruits, like apple, grape berry, olive, tomato and others, are described in this review. The need for environmentally valuable and potentially profitable technologies for the recovery, recycling and upgrading of residues from fruit processing is also discussed.

## Introduction

Through the ages, folk medicine has established the value of certain foods in human health maintenance. There is now mounting evidence that the healthiest diets are those loaded with plant foods. The health benefits ascribed to diets rich in fruit and vegetables include prevention, or at least reduction of the risk of chronic diseases occurring in the aging human population, such as cardiovascular disease, diabetes and cancer. In primary prevention, a diet rich in plant-derived food is the preferred treatment option for early type-2 diabetes and hypercholesterolemia (Hooper and Cassidy 2006). Such a diet can also lower the blood pressure, reduce the risk of heart disease and stroke, prevent some gastrointestinal problems like diverticulosis, and lower the risk of Alzheimer’s disease, cataracts and macular degeneration of the eye, as well as other age-related functional declines (Willet 1994; Liu 2003). There is considerable interest in bioactive compounds present in edible plants and thus in “letting your food be your medicine”, as recommended by Hippocrates.

Bioactive plant constituents that are being actively studied for their health enhancing potential include flavonoids, phenolic acids, carotenoids, tocopherols, alkaloids, lignans, tannins, salicylates and glucosinolates, plus another important class of secondary metabolites, the triterpenoids. Formed by the cyclization of the linear squalene molecule, these tetra- or pentacyclic compounds are distinguished by their wide structural diversity (Hill and Connolly 2011, and their previous annual reviews). Moreover, they are found in plants not only in a free form, but also as esters and glycoside conjugates called saponins. All forms of triterpenoids are widely distributed in edible and medicinal plants, and consequently, they are an integral part of the human diet.

Triterpenoids have been shown to possess numerous biological activities and display various pharmacological effects such as antiinflammatory, antiulcer, antibacterial, antiviral (including anti-HIV), hepatoprotective, immunomodulatory, hypolipidemic and cholesterol-lowering, antiatherosclerotic, wound-healing, anticoagulant and anticarcinogenic properties, combined with relatively low toxicity (Akihisa et al. 2001; Patočka 2003; Liu 2005; Dzubak et al. 2006; Sun et al. 2006; Jäger et al. 2009; Kuo et al. 2009; Rezanka et al. 2009; Wolska et al. 2010; Bishayee et al. 2011; Thoppil and Bishayee 2011). The multifunctionality of triterpenoids makes them promising multi-targeting agents in the treatment of certain cancers and inflammatory diseases. Due to their ability to act at various stages in the process of carcinogenesis, namely to block NF-κB activation, induce apoptosis and inhibit proliferation, invasion, metastasis and angiogenesis, these compounds may be considered for use in both chemoprevention and chemotherapy of cancer (Shishodia et al. 2003; Laszczyk 2009; Petronelli et al. 2009; Yadav et al. 2010).

Triterpenoids in their free and esterified forms are compounds with low polarity, and are therefore found in abundance in such plant parts as surface cuticle waxes and stem bark. Screens of various plant tissues to identify those that are rich in triterpenoids, point to fruit peels as a promising and highly available source material (Beindorff et al. 2001; Jäger et al. 2009).

## Fruit cuticle

The surfaces of aerial parts (leaves, flowers, fruits and non-woody stems) of all terrestrial plants are covered with a hydrophobic layer called a cuticle (Müller and Riederer 2005). The cuticle forms a protective coating to prevent desiccation of the plant organs due to uncontrolled non-stomatal water loss, as well as the loss of organic and inorganic compounds by leaching. The cuticle is the first protective barrier against abiotic and biotic environmental stresses; it protects the plant surfaces against mechanical damage, abrasions, infiltration of xenobiotics and potentially harmful irradiance, like UV-B radiation, and it is the first line of defense against infection by plant pathogens. This interface between the plant and its environment is relevant for colonization by epiphytic microorganisms and host recognition by insects and pathogenic fungi (Bringe et al. 2006). In agriculture, it is the target site for sprays including fertilizers, growth regulators, fungicides, insecticides and herbicides.

The cuticle has two main components, a structural matrix called cutin, and wax. Cutin is a polyester-type biopolymer, composed mainly of hydroxy- and hydroxyepoxy-fatty acids. Waxes are embedded in the cutin and form a continuous layer on its top, so that intracuticular and epicuticular wax layers can be distinguished (Jetter et al. 2000). Waxes extracted from diverse plant species consist of homologous series of very-long-chain aliphatics, i.e. fatty acids, aldehydes, primary and secondary alcohols, ketones, alkanes and alkyl esters. Triterpenoids, tocopherols or aromatic compounds may also be present in trace amounts in some species, while dominating the mixture in others. It has been reported that triterpenoids are located almost exclusively in the intracuticular wax compartment (Jetter and Schäffer 2001; Buschhaus and Jetter 2011).

The chemical composition of cuticular waxes shows great variability, not only among different plant species, but also between different organs of an individual plant, and is affected by the stage of plant development, its geographic location and the local environmental conditions. The cuticular wax layer forms an interactive flexible interface between the plant and its environment. Thus, the chemical composition of these waxes influences the morphology, arrangement and microstructure of the plant surface, which determines the relative adhesion of water, pesticides, fungal spores, and other airborne deposits (Belding et al. 2000). This means that cuticular waxes are responsible for the wettability and permeability properties of the cuticle. On the other hand, the thickness and texture of the cuticular wax can be affected by environmental factors such as relative humidity, soil moisture, sunlight and temperature. Thus, external conditions can significantly influence the amount and composition of cuticular wax, which can have an impact on the vitality of leaves and the quality of the fruits. In the case of fleshy fruits, in particular, the cuticle is an important factor contributing to shelf life and post-harvest storability. Variation in the cuticle composition may underlie differences in fruit resistance to desiccation and microbial infection (Kosma et al. 2010). The composition of the cuticle has been associated with the incidence and severity of fungal disease, particularly the presence of biologically active compounds which can reduce germination of conidia or inhibit germ tube growth of various fungi (Belding et al. 2000).

Compared with plant vegetative organs, especially leaves, far less information is available regarding the triterpenoid content of fruit cuticular waxes. Data concerning the triterpenoids contained in fruit cuticular waxes and their possible impact on the health benefits associated with fruit consumption, and the usefulness of the fruit peel as a source of such compounds, are presented in this review. Although not strictly botanical terms, “peel” and sometimes “skin” are used to denote the outer fruit layers, composed of the cuticle and multiple cell types, including epidermis, collenchyma, and sometimes even parenchyma, depending on how the peels are removed. The term “flesh” refers to the pericarp material from which the peel has been removed; this tissue is predominantly composed of parenchyma and collenchyma (Mintz-Oron et al. 2008).

## Apple

Among all edible fruits, apples (*Malus pumila* Mill., *Malus sylvestris* L. (Mill.) var. *domestica* Borkh.) have one of the longest history of being significant part of the human diet and they also have proven medicinal properties (Cefarelli et al. 2006). The proverb “an apple a day keeps the doctor away” has been validated by numerous observations. Current annual global apple production is approximately 70 million tonnes (McGhie et al. 2012). The consumption of apples has been linked to the prevention of various chronic diseases and is believed to reduce the incidence of lung cancer, cardiovascular disease, symptoms of chronic obstructive pulmonary disease, and the risk of thrombotic stroke (He and Liu 2007). Apple peel exhibits more potent antioxidant and antiproliferative activity than apple flesh (Wolfe et al. 2003; Wolfe and Liu 2003), which suggests that the peel contains the majority of the bioactive phytochemicals.

### Triterpenoid composition of apple peel

Epicuticular waxes of apples were studied as early as 1920, and ursolic acid was one of the first reported components (Belding et al. 1998; Verardo et al. 2003; Rudell et al. 2009). Apple peel has since been frequently recommended as a source of ursolic acid that may be utilized for various purposes (Beindorff et al. 2001; Glinsky and Branly 2001; Jäger et al. 2009). Several methods have been devised for the isolation of this compound from apple, including extraction of fresh homogenized peels with ethyl acetate (with a yield of 0.15 % of fresh weight of peel obtained from the Red Delicious variety, He and Liu 2007), extraction of dried peel with chloroform (0.7 % of peel of the Fuji variety, Yamaguchi et al. 2008), and accelerated solvent extraction (ASE) of dried peel with ethyl acetate (with a yield ranging from 0.2 to 2.1 % for 11 cultivars tested; Jäger et al. 2009). Besides various techniques of adsorption chromatography and HPLC, high speed counter-current chromatography (HSCCC) has also been used to purify this compound (Frighetto et al. 2008).

**Fig. 1:**
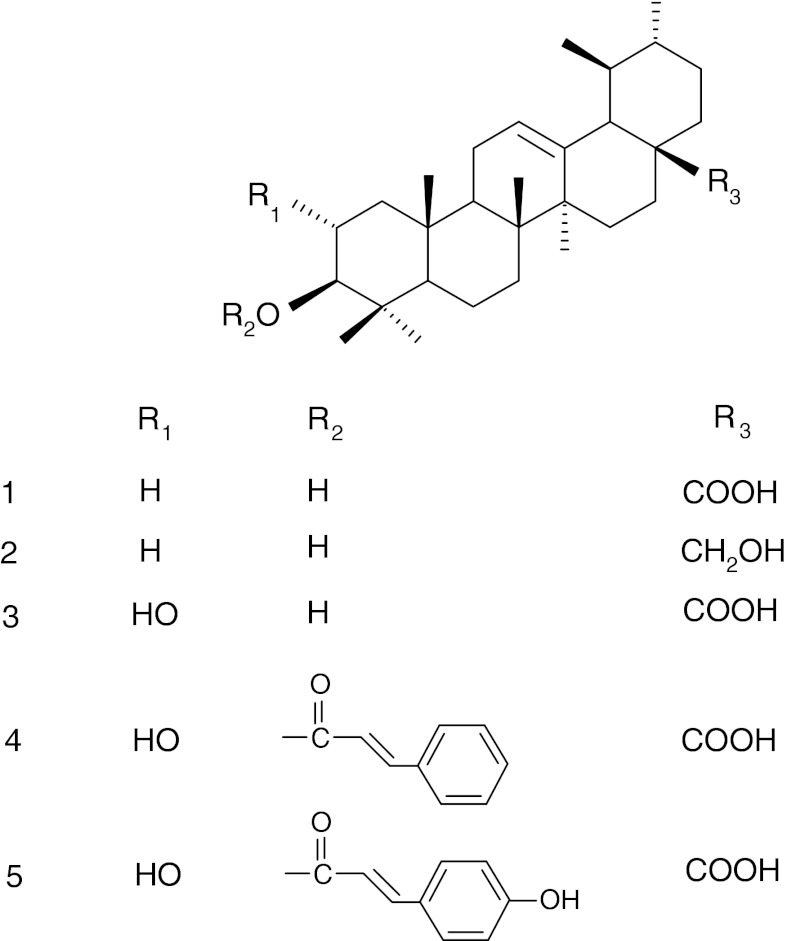
Structures of some triterpenoids occurring in apple (*M. pumila*) fruit cuticular waxes: ursolic acid (*1*), uvaol (*2*), 2α-hydroxyursolic acid (*3*), 3β-*trans*-cinnamoyloxy-2α-hydroxyurs-12-en-28-oic acid (*4*), 3β-*trans*-*p*-coumaroyloxy-2α-hydroxyurs-12-en-28-oic acid (*5*)

The occurrence of ursolic acid in apple peels is well documented, but there is a growing list of other triterpenoids that are also present in apple fruit cuticular wax and have yet to be fully investigated. Ma et al. (2005) reported the isolation of six triterpenoid compounds with ursane and oleanane skeletons (ursolic acid, 2α-hydroxyursolic acid, euscaphic acid, 2α,3α-dihydroxy-urs-12-en-28-oic acid and 2α,3α-dihydroxy-olean-12-en-28-oic acid, uvaol) in an ethyl acetate extract of the peel of apples purchased from a supermarket in Beijing, China (Table 1). The occurrence of oleanolic acid (in addition to ursolic acid and uvaol) in the Holsteiner Cox variety was described by Ellgardt (2006), and β-sitosterol was found by Verardo et al. (2003) in the cultivars Florina, Golden B and Ozark Gold. Thirteen triterpenoids were isolated and identified by He and Liu (2007) in the peel of Red Delicious apples (Table 1), with ursolic acid, 2α-hydroxyursolic acid, 3β-*trans*-cinnamoyloxy-2α-hydroxyurs-12-en-28-oic acid, 3β-*trans*-*p*-coumaroyloxy-2α-hydroxyurs-12-en-28-oic acid (Fig. 1) and 3β-*trans*-*p*-cinnamoyloxy-2α-hydroxyolean-12-en-28-oic acid being the most abundant compounds. Nine ursane (including annurcoic and annurconic acids, compounds described for the first time), two oleanane and two lupane triterpenes, along with two steroids were isolated and identified in whole apples of the Annurca cultivar (Cefarelli et al. 2006). A recent comprehensive analysis of triterpene acids in seven apple cultivars from New Zealand performed using UHPLC–HRMS, demonstrated the presence of multiple isomeric compounds of the ursane-type acids (oxo, hydroxyl, dihydroxy, trihydroxy, etc.) (McGhie et al. 2012).

**Table 1:** Triterpenoid profile of cuticular waxes of some edible fruits

Fruit	Triterpenoid profile	Amount	Predominant compounds	Method of wax extraction	Method of triterpenoid identification	Reference
Apple *(Malus pumila* Mill*.)*	Euscaphic acid; 2α,3α-dihydroxy-olean-12-en-28-oic acid; 2α,3α-dihydroxy-urs-12-en-28-oic acid; 2α-hydroxyursolic acid; ursolic acid, uvaol	77 % of the precipitate obtained from the peel extract	Ursolic acid (98 % of triterpenoid mixture)	Extraction (supersonication) of fresh peel with ethyl acetate	NMR	Ma et al. (2005)
Apple *(Malus domestica* Borkh.) cv. Holsteiner Cox	Oleanolic acid; ursolic acid; uvaol	0.34–0.42 % of peel d.w.	Ursolic acid (0.28–0.34 % of peel d.w.)	Extraction of freeze-dried peel with ethanol (after preliminary extraction with n-hexane)	HPLC (R_T_)	Ellgardt (2006)
Apple *(M. pumila* Mill*.)* cv. Red Delicious	3β-*cis*-*p*-coumaroyloxy-2α-hydroxyolean-12-en-28-oic acid; 3β-*cis*-*p*-coumaroyloxy-2α-hydroxyurs-12-en-28-oic acid; 3β,28-dihydroxy-12-ursene; 3β,13β-dihydroxyurs-11-en-28-oic acid; 2α-hydroxy-3β-{[(2*Z*)-3-phenyl-1-oxo-2-propenyl]oxy}olean-12-en-28-oic acid; 2α-hydroxyursolic acid; maslinic acid; 3β-*trans*-cinnamoyloxy-2α-hydroxyurs-12-en-28-oic acid; 3β-*trans*-*p*-cinnamoyloxy-2α-hydroxyolean-12-en-28-oic acid; 3β-*trans*-*p*-coumaroyloxy-2α-hydroxyolean-12-en-28-oic acid; 3β-*trans*-*p*-coumaroyloxy-2α-hydroxyurs-12-en-28-oic acid; 2α,3β,13β-trihydroxyurs-11-en-28-oic acid; ursolic acid	0.15 % of the mass of fresh peels, 19.5 % of the peel extract	Ursolic acid (0.15 % of the mass of fresh peels, 18 % of the peel extract)	Extraction of homogenized fresh peel with acetone and ethyl acetate	NMR	He and Liu (2007)
Grape berry (*Vitis vinifera* L.) cv. Cabernet Sauvignon	Oleanolic acid; oleanolic aldehyde; β-sitosterol; β-sitosterol-3-*O*-β–D-glucoside; β-sitosterol-6′-linolenoyl-3-*O*-β-D-glucopyranoside	0.075 % of fresh skin mass	Oleanolic acid (86 % of triterpenoid mixture)	Extraction of blended skin with methanol and ethyl acetate	NMR	Zhang et al. (2004)
Grape berry (*V. vinifera* L.)	Oleanolic acid; β-sitosterol; β-sitosterol-3-*O*-β–D-glucoside	0.027 % of fresh berry mass in cv. Othello	Oleanolic acid (0.003–0.016 % of fresh berry mass)	Extraction of detached and homogenized skin with ethyl acetate	LC–MS	Orbán et al. (2009)
Olive (*Olea europaea* L.) cv. Coratina	α-amyrin; β-amyrin; betulinic acid; erythrodiol; maslinic acid; oleanolic acid; β-sitosterol, stigmasterol, uvaol	52 and 26 % of the total wax extract in green and black fruit, respectively	Oleanolic acid (70 and 83 % of total triterpenoids in green and black fruit, respectively)	Immersion of whole fruits in chloroform	GC–MS	Bianchi et al. (1992)
Olive (*O. europaea* L.) cv. Arbequina	Maslinic acid; oleanolic acid	0.23 and 0.19 % of fruit d.w. in green and black fruit, respectively	Maslinic acid (0.18 and 0.15 % of fruit d.w. in green mature and black ripe fruit, respectively)	Extraction of mechanically obtained epicarp with absolute ethanol	GC (R_T_)	Guinda et al. (2010)
Tomato (*L. esculentum* L.)	α-amyrin; β-amyrin; δ-amyrin; bauerenol; cycloartenol; germanicol; lupeol; multiflorenol; β-sitosterol; stigmasterol; taraxasterol; ψ-taraxasterol; taraxerol	13.7 % of the total wax extract (average from 26 cultivars)	δ-amyrin (5.6 % of wax extract, i.e. 41.2 % of total triterpenoids); β-amyrin (3.2 % of wax extract); α-amyrin (3 % of wax extract)	Dipping of whole fruits into *tert*-butylmethyl ether/methanol (9:1) in ultrasonic bath	GC–MS	Bauer et al. (2004a, b)
Tomato (*L. esculentum* L.) cv. MicroTom (wild-type)	α-amyrin; β-amyrin; β-amyrin derivative; δ-amyrin; cholesterol; lanosterol; lupeol derivative I; multiflorenol; β-sitosterol; stigmasterol; taraxasterol; ψ-taraxasterol; taraxerol	21 % of the total wax extract (in mature fruit)	α-, β-, δ-amyrins (76-91 % ot total triterpenoids)	Extraction of enzymatically isolated cuticle with chloroform	GC–MS	Leide et al. (2007)

*GC*–*MS* gas chromatography–mass spectrometry, *HPLC* high performance liquid chromatography, *LC*–*MS* liquid chromatography–mass spectrometry, *NMR* nuclear magnetic resonance, *R*_*T*_ retention time

Apples are a very good example of how the quantity and composition of epicuticular wax can vary between cultivars (Belding et al. 1998; Belding et al. 2000; Verardo et al. 2003). For example, among twelve cultivars tested, the triterpenoid content ranged from 32 % of cuticular wax extract obtained from Royal Gala apples to almost 70 % in Pure Gold fruit (Belding et al. 1998). The amounts of ursolic acid, oleanolic acid and uvaol were higher by 17, 15 and 29 %, respectively (Table 1), in cuticular wax of Holsteiner Cox apples originating from integrated production (restricting the use of pesticides and fertilizers to a required minimum) compared with those produced organically (production based on recycling and local renewable resources with a ban on the use of chemical pesticides and commercial fertilizers), which indicates that the method of cultivation can influence the apple peel composition (Ellgardt 2006). The triterpenoid content of apple peel can also be significantly affected by post-harvest conditions including cold storage of fruit, as was revealed by studies on metabolomic changes associated with oxidation during the development of superficial scald symptoms (Rudell et al. 2009). In turn, cuticular wax composition can influence the quality of fruits during storage and their shelf life. For example, Golden Delicious apples lose water more rapidly than other cultivars in storage, which is commonly thought to be due to the smaller wax mass (Belding et al. 1998). At the same time, the cuticular wax of these apples contains the highest proportion of triterpenoids (54–70 % of total wax extract), which might explain the poor water retention of this cultivar, since a high level of triterpenoids in cuticular wax is not positively correlated with water impermeability. The composition of the cuticular wax of apples has also been studied with regard to its possible relevance in the avoidance of fruit damage caused by insects and pathogens such as *Peltaster fructicola* and *Leptodontidium elatius*, although the relative proportions of triterpenoids were not found to be related to the severity of sooty blotch disease caused by these fungi (Belding et al. 2000).

Apple peels are a waste product of juice and canned fruit production, and their utilization to generate a value-added food ingredient can be economically beneficial (Wolfe and Liu 2003; Djilas et al. 2009). However, there have been no studies on the consequences of the commercial practice of waxing apples on the occurrence of triterpenoids in apple peel. Prior to packaging of apple fruits, they are washed by scrubbing the surface to remove field dust and chemical residues, which removes approximately 50 % of the natural wax coating. To replace the original protective layer, supplementary waxes (usually carnauba or shellac, which do not contain ursolic acid) are applied to the surface of apples. However, as triterpenoids are most likely to be localized in the deeper layers of plant cuticular waxes, i.e. intracuticular wax substructure (Buschhaus and Jetter 2011), it is possible that this treatment does not remove all these compounds from the peel of packed and waxed apples sold in supermarkets.

### Pharmacological properties of triterpenoids found in apple peel

A diet enriched in apples has been associated with the reduced incidence of many chronic diseases, including lung cancer (Wolfe and Liu 2003). The antitumor activity of triterpenoids isolated from apple peel has been investigated with a variety of tests. Ursolic acid and 2α-hydroxyursolic acid exhibited in vitro growth inhibitory activity against four tumor cell lines, HL-60, BGC, Bel-7402, and HeLa, with ED_50_ values ranging from 45 to 72 μg/ml (Ma et al. 2005). Moreover, ursolic acid can serve as a precursor of more potent cytotoxic derivatives, such as the 3-amino and 28-aminoalkyl compounds, the former of which was shown to be 20-times more potent than the parent compound (Ma et al. 2005). Most of the triterpenoids, including their *p*-coumaryloxy-esters, isolated from apple peel of the Red Delicious variety by He and Liu (2007), showed potent antiproliferative activities against human cell lines of HepG2 liver cancer, MCF-7 breast cancer and Caco-2 colon cancer. Yamaguchi et al. (2008) reported a slight harmful effect of ursolic acid isolated from apple peel of the Fuji variety on normal mouse embryo cells (growth inhibition by 7 % at an ursolic acid concentration of 10 μM), which contrast with its efficacy in suppressing the growth (by 82 %) of highly metastatic tumorigenic cells. However, higher concentrations of ursolic acid can also be harmful to healthy cells: a concentration of 20 μM suppressed the growth of more than 90 % of tumorigenic cells, but it also affected about 60 % of normal cells.

Ursolic acid itself is ubiquitous in medicinal and edible plants (e.g. herbs such as basil, peppermint, rosemary), and it has attracted attention due to its diverse pharmacological properties including antitumorigenic effects exerted by inhibition of the STAT3 pathway. This compound was shown to inhibit NF-κB activation induced by carcinogenic agents through suppression of IκBα kinase and p65 phosphorylation, and it also suppressed TNF-induced expression of cyclin D1, cyclooxygenase 2 (COX-2) and matrix metalloproteinase 9, which are involved in the initiation, promotion and metastasis of tumors (Shishodia et al. 2003).

## Grape berry

Grapevine (*Vitis vinifera* L.), which is native to southern Europe and Western Asia, is cultivated also on other continents (in addition to North American grapevine species like *V. labrusca, V. rotundifolia*), and its fruit is highly valued for its great economic importance and many health benefits. In the context of the Mediterranean dietary tradition, grape consumption is linked to reduction of the incidence of chronic illnesses, such as cancer, cardiovascular diseases, ischemic stroke, neurodegenerative disorders and aging (Iriti and Faoro 2009; Yadav et al. 2009; Ali et al. 2010). Grapevine products (fresh berries, raisins, juice, wine) are well known for their antioxidant content, and therefore, grape extracts are also widely used in various cosmetic formulas. Many studies have shown that active components, including polyphenols, resveratrol, hydroxytyrosol and recently melatonin, may contribute to the health benefits associated with regular consumption of grape products (Leifert and Abeywardena 2008; Ali et al. 2010; Vislocky and Fernandez 2010; Fernández-Mar et al. 2011). However, relatively few studies have focused on the presence and health promoting properties of triterpenoids from grape berries.

### Triterpenoid composition of grape berry skin

Some preliminary investigations of the composition of grape berry wax were carried out as early as 1892, and by 1938, oleanolic acid and β-sitosterol had been identified (Radler and Horn 1965). Oleanolic acid was the main constituent of chloroform extracts of cuticular waxes from the fruits of the sultana vine as well as those of several American *Vitis* species and hybrids (Radler 1965a, b; Radler and Horn 1965). The amount of oleanolic acid in wax extract of young and mature sultana vine fruits ranged from 45 to 65 %, respectively, and accounted for 50 % of the total wax extract obtained from dried grapes (Radler and Horn 1965; Radler 1965a). The occurrence of remarkable amounts of oleanolic acid in grape berry wax was confirmed in many subsequent studies performed on various grape cultivars. This compound was reported to constitute 50–80 % of the total weight of wax extracts obtained from fruits of several Japanese grape varieties at the harvest stage (Yamamura and Naito 1983), and it was the main component of grape berry cuticular waxes of three clones of Pinot noir (from Champagne vineyards) at all stages of fruit development (Comménil et al. 1997). Furthermore, a comparison of twelve grape varieties (five white and seven red varieties originating from the Hungarian Eger and Alföld wine regions) revealed significant variation in the oleanolic acid content, ranging from 31 to 162 mg/kg of fresh berry mass calculated for fruit wax, or from 157 to 239 mg/kg of fresh berry mass when calculated for whole fruit skins (Orbán et al. 2009).

**Fig. 2:**
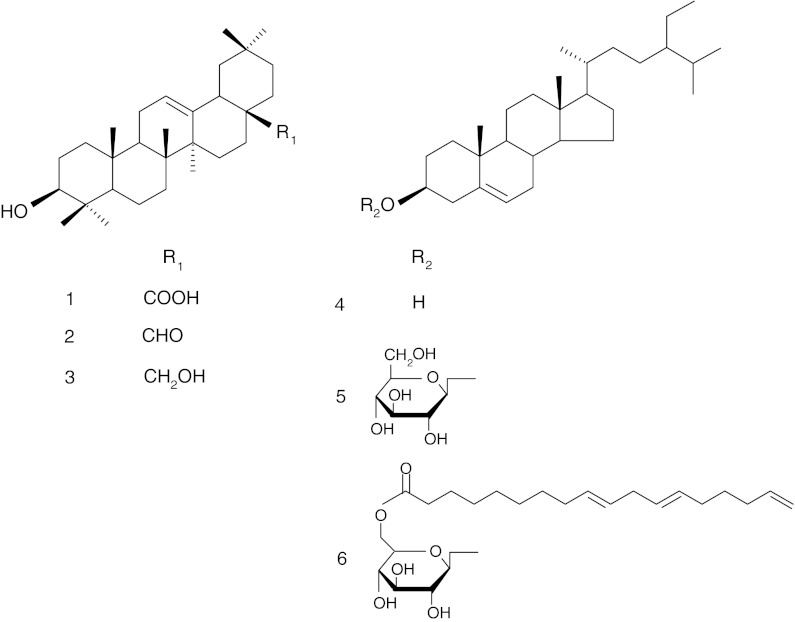
Structures of some triterpenoids occurring in grape berry (*V. vinifera*) fruit cuticular waxes: oleanolic acid (*1*), oleanolic aldehyde (*2*), erythrodiol (*3*), β-sitosterol (*4*), β-sitosterol-3-O-β–D-glucoside (*5*), β-sitosterol-6′-linolenoyl-3-O-β-D-glucopyranoside (*6*)

Besides oleanolic acid, other triterpenoids have been identified in grape berry cuticular wax, namely oleanolic aldehyde (Dagna et al. 1982; Zhang et al. 2004), erythrodiol (Dagna et al. 1982) (Fig. 2) and a group of phytosterols and their derivatives (Dagna et al. 1982; Le Fur et al. 1994; Zhang et al. 2004; Orbán et al. 2009). The phytosterols β-sitosterol, campesterol and stigmasterol were detected in chloroform extracts of the dried skin of berries obtained from fourteen grape varieties of the Piedmont region (Dagna et al. 1982). Subsequently, lanosterol was identified in Folch mixture (chloroform:methanol 2:1, v/v) extracts from lyophilised skins of Chardonnay grape berries from Burgundy (Le Fur et al. 1994). The level of phytosterols exhibited a characteristic pattern of fluctuation: increasing at peak maturity and decreasing during the last stage of ripening. The predominant steroid compound in the grape berry cuticular wax was β-sitosterol, accounting for 0.1 % of dry berry skin and representing 86–89 % of the total detected phytosterols. The levels of stigmasterol and campesterol were estimated at 85 and 43 μg/g of dry weight, respectively, while lanosterol was found in much lower amounts (Le Fur et al. 1994). β-Sitosterol and its monoglycosidic derivative, β-sitosterol-3-*O*-β-D-glucoside (Fig. 2) were identified by liquid chromatography–mass spectrometry (LC–APCI–MS) in ethyl acetate extracts of berry skins of grapes from the Eger and Alföld regions (Table 1), with amounts ranging among varieties from 12 to 73 mg/kg of fresh berry mass for β-sitosterol, and 2–13 mg/kg of fresh berry mass for its monoglucoside (Orbán et al. 2009). In addition to β-sitosterol-3-*O*-β–D-glucoside, another steroid derivative, β-sitosterol-6′-linolenoyl-3-*O*-β-D-glucopyranoside (Fig. 2) was found in methanol extracts of blended skins of Cabernet Sauvignon grape berries harvested from Michigan State University Horticulture farm (Table 1, Zhang et al. 2004). Studies examining Thompson seedless raisins (Rivero-Cruz et al. 2008) and grape pomace from the red Sicilian cultivar Nerello Mascalese (Amico et al. 2004), identified the compounds betulin, betulinic acid, lupeol, oleanolic acid acetate and β-sitosterol glucoside peracetate, which expanded the list of triterpenoids found in grape berry cuticular waxes.

### Participation of triterpenoids in the spatial arrangement of grape berry wax

The grape berry cuticle is characterized by a high content of waxes (about 25 % of cuticular weight) and a high degree of molecular order (Casado and Heredia 1999, 2001). Oleanolic acid, although highly abundant in this layer (up to 60 %, or even more, of the total wax) appears not to contribute to the ordered crystalline structure of the cuticle. A molecular model of the spatial arrangement of oleanolic acid and the main aliphatic compound, n-hexacosanol, has been proposed to explain the organization of the grape berry cuticle. Oleanolic acid can form a dimer via hydrogen bond interactions between the hydroxyl group of one molecule and the carboxyl group of a second molecule. The remaining functional groups may interact with complementary groups of other molecules, including aliphatic alcohols, forming a tri-dimentional arrangement (Casado and Heredia 1999). The ultrastructure and chemical composition of grape cuticular waxes may also play an important role in the resistance of grape berries to *Botrytis cinerea*, although a direct relationship between the triterpenoid content and susceptibility to bunch rot disease caused by this pathogen has yet to be confirmed (Comménil et al. 1997).

### Pharmacological properties of triterpenoids found in grape berry skin

The high abundance of oleanolic acid in grape berry cuticular wax can contribute to health benefits ascribed to grape consumption. Like its isomer ursolic acid, oleanolic acid is known to have numerous pharmacological properties including anticancer, anti-inflammatory, antidiabetogenic, antimicrobial, hepato- and cardioprotective, anti-HIV and anti-multiple sclerosis effects (Liu 2005; Yeung and Che 2009, Martín et al. 2010). Oleanolic acid isolated from Cabernet Sauvignon grape skin was shown to be capable of in vitro regulation of insulin secretion, significantly stimulating insulin production at concentrations ranging from 6 to 50 μg/ml, in a dose-dependent manner, similarly to glucose. Oleanolic aldehyde was also able to influence insulin secretion in vitro. Furthermore, oleanolic acid applied to cells at a concentration of 100 μg/ml inhibited COX-2 enzyme activity by 10 %. These findings suggest that the consumption of whole grape berries may help to reduce the incidence of type-2 diabetes and inflammation (Zhang et al. 2004). In China, *V. vinifera* berries called suosuo grapes, produced in the Turpan, Shanshan and Hetian regions of Xinjiang, have been used in Uighur folk medicine for the prevention and treatment of liver disorders. In traditional medicine, these grapes have also been used to treat diarrhea, hepatitis and stomach aches. The grape berry triterpenoid fraction, containing mainly oleanolic acid, exhibited protective effects against immunological liver damage in vitro (Liu et al. 2007). This hepatoprotective activity was later confirmed by in vivo experiments in mice (Liu et al. 2010). Oleanolic acid and oleanolic aldehyde obtained from Thompson seedless raisins displayed significant antimicrobial activity (MIC values of 250–625 μg/ml) against *Streptococcus mutans* and *Porphyromonas gingivalis*, two oral pathogens most commonly associated with human dental carries and periodontal diseases (Rivero-Cruz et al. 2008). The growth of these pathogens was also inhibited by other triterpenoid compounds occurring in raisins, such as betulin, betulinic acid, β-sitosterol and β-sitosterol glucoside. The potential benefits to oral health and disease prevention due to the presence of antimicrobial triterpenoids, means that raisins may represent a healthy alternative to widely consumed sugary snack foods (Wu 2009). Moreover, β-sitosterol and its glucoside possess a variety of beneficial properties and were shown to have serum cholesterol lowering, cancer preventative, antimutagenic and anti-inflammatory activities (Awad and Fink 2000; Piironen et al. 2000; Villasenor et al. 2002). Lupeol, another triterpene compound isolated from grape pomace (Amico et al. 2004), is also known for its significant pharmacological properties, including anticancer, antiprotozoal, chemopreventive and anti-inflammatory activities (Gallo and Sarachine 2009).

Numerous health benefits, including protection against heart disease, are ascribed to the moderate consumption of wine, one of the most popular grape berry products. A worldwide monitoring system for cardiovascular diseases organized by the World Health Organization showed that the mortality rate from coronary disease is much lower in France than in other industrialized countries despite similar consumption of saturated fats (World Health Statistics Annual 1989). This phenomenon, called the French paradox, has mostly been explained by the high wine intake in France, and is usually linked to the cardioprotective properties of phenolics (Frankel et al. 1993). There have been few studies examining triterpenoid compounds occurring in wine. The triterpene composition of five red wines, i.e. Bordeaux Graves 2000, Bordeaux Haut médoc 1998, Bordeaux Pessac Léognan 1999, Burgundy 1996 and 2000, and two white wines, i.e. Bordeaux Graves 1999 and 2000, was compared and a considerable qualitative variation was observed. 2α,3β,19α-Trihydroxyolean-12-ene-24,28-dioic acid and 2α,3β,19α,23-tetrahydroxyolean-12-ene-24,28-dioic acid were found in every wine tested except Burgundy 1996. 28-β-D-Glucopyranosyl-trihydroxyolean-12-ene-24,28-dioic acid was identified in Bordeaux Haut Médoc 1998, Bordeaux Pessac Léognan 1999 and Bordeaux Graves 2000, while 28-β-D-glucopyranosyl-tetrahydroxyolean-12-ene-24,28-dioic acid was found only in Burgundy 1996 and Bordeaux Graves 2000. Although the identification of triterpenoids occurring in wine is very interesting, this study also underlined the fact that some of these compounds could come from the oak wood barrels used during wine maturation (Aramon et al. 2003). This was recently confirmed by the detection in wine of two novel compounds, quercotriterpenoside I and II: oleanane-type triterpenes substituted with galloyl and glucosyl residues that originate from oak barrels (Marchal et al. 2011). Grape pomace, which is estimated to represent 13 % of the grape by weight, is a significant by-product of the process of wine-making and a promising source of useful compounds: not only polyphenols, but also biologically active triterpenoids. For example, a freeze-dried grape pomace macerate of red grape Nerello Mascalese was defatted with n-hexane and extracted with ethyl acetate to obtain oleanolic acid, lupeol and β-sitosterol 3-*O*-glucoside with yields of 0.1575 %, 0.0085 % and 0.0249 % of pomace dry weight, respectively (Amico et al. 2004).

## Olive

Olive (*Olea europaea* L.) is a fruit of major agricultural importance in the Mediterranean region. Olive tree cultivation started 6,000 years ago and thus the olive is the oldest cultivated tree. Olive oil, one of the basic components of the traditional Mediterranean diet, has become increasingly popular due to its beneficial nutritional and medicinal properties, including reduction of the risk of coronary heart disease and atherosclerosis, the prevention of several types of cancer and modification of the immune and inflammatory responses (Owen et al. 2004; Ortega 2006). However, while the beneficial effects of the monounsaturated fatty acids and phenols in olive oil are well recognized, much less attention has been paid to other compounds, including triterpenoids (Stiti and Hartmann 2012).

### Triterpenoid composition of the olive skin

**Fig. 3:**
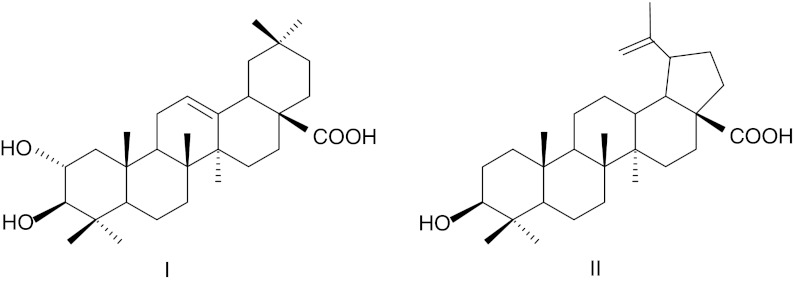
Structures of some triterpenoids occurring in olive (*O. europaea*) fruit cuticular waxes: **I** maslinic acid, **II** betulinic acid

The presence of the pentacyclic triterpene acids, maslinic (Fig. 3) and oleanolic, in olive fruits has long been known (Vioque and Maza 1963; Bianchi et al. 1992; Pérez-Camino and Cert 1999). These compounds were identified as the major components of the chloroform-soluble waxes of olive fruits of the Italian cultivar Coratina collected in Pescara, accounting for 26 and 38 % of the total wax of green mature and black ripe olives, respectively. This study also revealed differences between green and black olives in the content of the dihydroxy alcohols, erythrodiol and uvaol, which were present in substantial amounts (14 %) in the wax of green olives, but only in traces in that of black olives. In addition, small amounts of betulinic (Fig. 3) and ursolic acids, α- and β-amyrins, as well as phytosterols, mainly β-sitosterol and stigmasterol, were detected (Table 1) (Bianchi et al. 1992). Significant amounts of triterpene acids in olive cuticular waxes (maslinic and oleanolic acid representing 55–68 % and 31–44 % of total wax extract, respectively) were also reported for cutivars Cipressino, Dritta and Leccino (Bianchi et al. 1994). The formation of triterpenoids throughout fruit ontogeny at 13 distinct stages of development over a period of 33 weeks after flowering was studied in the olive cultivar Chemlali by Stiti et al. (2007). Numerous tetra- and pentacyclic triterpenes and sterols were identified in a dichloromethane/methanol (2:1) extract of entire olive fruit, including compounds of different carbon skeletons: oleanane, ursane, lupane, taraxane, euphane, baccharane and lanostane. At the onset of fruit development, between 12 and 18 weeks after flowering, high amounts of the triterpene monols (α- and β-amyrins as well as 28-nor-α- and 28-nor β-amyrins; 0.043 % of fruit dry weight, 13 % of all triterpenoids) as well as more oxygenated compounds, such as triterpene diols (mainly erythrodiol and uvaol; 0.07 % of d.w., 21 % of triterpenoids) and acids (3-*epi*-betulinic, oleanolic, maslinic and ursolic; 0.21 % of d.w., 66 % of triterpenoids), were detected. From 21 weeks after flowering, when the olive fruit reached its final size and began to turn from green to purple, α- and β-amyrins were no longer present, whereas the class of methylsterols started to be formed. The level of diols decreased to 0.002 % of d.w. (0.5 % of triterpenoids), while the content of triterpene acids reached a peak of almost 0.4 % of d.w. (99 % of total triterpenoids). In the last stage of fruit development, sterol end products were progressively accumulated in larger amounts, while the level of triterpene acids decreased to 0.25 % of d.w. During the earlier phases of fruit growth, oleanolic acid was the predominant triterpenoid, followed by maslinic acid; whereas in ripe olives, maslinic acid was the most abundant. In the 12th week after flowering, oleanolic acid accounted for 40 % of total triterpenoids and maslinic acid represented 26 %. Subsequently this ratio changed, and by the 30th week after flowering, the level of oleanolic acid had decreased to 38 %, while maslinic acid had increased to 61 % of total triterpenoids. Thus, the profile and the content of different triterpenoids in olive fruit seemed to be significantly influenced by the fruit developmental stage. This finding was confirmed for the three main Spanish olive cultivars, Picual, Hojiblanca and Arbequina, harvested in La Rinconada (Seville). The total triterpene content was higher in the unripe fruits (0.21–0.23 % of fruit d.w., depending on the cultivar) and diminished as ripening proceeded, with a decrease of 20 % between the green mature and the black ripe stages (final content 0.17–0.19 % of d.w). The ratio of maslinic and oleanolic acids was also dependent on the olive cultivar, with levels of the former 2.5-fold higher than those of the latter in fruits of the Pictual and Hojiblanca cultivars, and four fold higher in Arbequina. A study of the distribution of pentacyclic triterpenoids among Arbequina olive fruit tissues revealed that maslinic and oleanolic acids were exclusively found in the epicarp, which pointed to the cuticular waxes as the main location of these compounds in the olive fruit (Table 1, Guinda et al. 2010).

The level of triterpenoids in olives that are sold in the market can be significantly influenced not only by the cultivar or the stage of fruit ripeness, but also by the method of processing. Higher concentrations of maslinic and oleanolic acids were found in olives that had not been treated with alkali (turning colour olives, natural black olives), whereas pitting and stuffing did not significantly affect the level of these compounds. Treatment with NaOH, which is employed to debitter the fruits, was shown to cause the loss of triterpene acids due to their solubilization (after sodium salt formation) in alkaline solutions (Romero et al. 2010).

### Pharmacological properties of triterpenoids found in the olive skin

Olive triterpenoids have been attributed numerous health-promoting properties, such as anticancer (Martín et al. 2009; Juan and Planas 2010), antihyperglycemic (Liu et al. 2011) and antiparasitic activities (De Pablos et al. 2010; Moneriz et al. 2011). An extract from the skin of olive fruits, composed mainly of maslinic (73 %) and oleanolic (26 %) acids, inhibited proliferation and induced apoptosis in HT-29 human colon cancer cells in vitro (Juan et al. 2006). Subsequent studies on the activities of individual compounds revealed that oleanolic acid had a moderate antiproliferative effect on HT-29 cells (EC_50_ of 160 μmol/l) and simultaneous moderate cytotoxicity at concentrations higher than 250 μmol/l, whereas maslinic acid inhibited cancer cell growth with an EC_50_ of 101 μmol/l without necrotic effects. Oleanolic acid failed to activate caspase-3, a prime apoptosis protease, whereas maslinic acid increased the activity of this enzyme by 3, 3.5 and five fold at non-toxic concentrations of 10, 25 and 50 μmol/l, respectively. Thus, maslinic acid was proposed as a promising new compound for the chemoprevention of colon cancers (Juan et al. 2008). An in vivo study on hyperlipidemia induced in rats by a high cholesterol diet, revealed that posterior supplementation with olive pomace extracts rich in maslinic and oleanolic acids significantly modulated the consequent high levels of serum cholesterol and triglycerides, suggesting the possibility of utilizing olive pomaces for the prevention and treatment of hyperlipidemia (Liu et al. 2011). The triterpenoids occurring in olive pomace oil, maslinic and oleanolic acids as well as erythrodiol and uvaol, induced vasorelaxation in the aorta of hypertensive rats (Rodriguez-Rodriguez et al. 2006). Erythrodiol and uvaol were also found to be potent inhibitors of the proliferation of human 1321N1 astrocytoma cells in vitro, suggesting that these triterpenoids, which are capable of crossing the blood–brain barrier, may have the potential for use in prevention and treatment of brain cancers. It has been suggested that the induction of apoptosis by these compounds is mediated by the activation of a ROS/JNK (reactive oxygen species/c-Jun N-terminal kinase) pathway (Martín et al. 2009).

Several studies have indicated that maslinic acid is a potent antiparasitic agent. When applied to erythrocytic cultures, maslinic acid hindered the maturation of *Plasmodium falciparum* (the pathogen causing malaria) from the ring to the schizont stage, and could thus prevent the release of merozoites and the subsequent invasion. Maslinic acid effectively inhibited the proteolytic processing of the merozoite surface protein complex as well as plasmodial metalloproteases of the M16 family. Moreover, several others putative targets of maslinic acid have been suggested, such as phospholipase A2. The identification of maslinic acid as a potential multi-target drug against the intra-erythrocytic cycle of *Plasmodium* may be highly significant for the treatment of malaria, because most antimalarials target only a single process of the parasite infective cycle, which favours the appearance of resistant mutants (Moneriz et al. 2011). Maslinic acid was also proposed as a new natural coccidiostatic therapy against *Eimeria tenella*, a parasite causing coccidiosis in chickens (*Gallus domesticus*), which has a considerable economic impact on poultry production. The anticoccidial index (ACI), expressing the release of oocysts estimated at 10 days post-infection, was higher in chickens given maslinic acid (210) than in those receiving sodium salinomycin (173), the conventional coccidiostatic treatment. Furthermore, maslinic acid was also observed to affect the gliding of *Toxoplasma gondii* (De Pablos et al. 2010).

Due to the localization of olive triterpenoids in fruit cuticular waxes, olive oil, the most popular olive fruit product, contains only small amounts of these compounds (Pérez-Camino and Cert 1999; Stiti and Hartmann 2012). The concentration of maslinic and oleanolic acids depends on the olive cultivar and the oil quality: extra virgin oils contain <200 mg/kg of these triterpenoids, their content exceeds 300 mg/kg in virgin oil, and crude pomace oils have up to 10 g/kg of these compounds, although the latter oils have to be refined before consumption (Pérez-Camino and Cert 1999; Romero et al. 2010). “Orujo” or “alpeorujo” olive oil, as it is called in Spain, obtained from the pomace remaining after the mechanical extraction of virgin oil and subsequent centrifugation of the olive paste, represents a rich source of biologically active triterpenoids (Rodriguez-Rodriguez et al. 2006; García et al. 2008). The olive oil industry generates large amounts of various wastes, which contain many important biologically active compounds, and there is growing interest in their recovery (Niaounakis and Halvadakis 2006; Fernández-Bolaños et al. 2006). For example, maslinic acid obtained from the solid waste from olive-oil production was successfully applied as a feed additive to stimulate growth and hepatic protein-turnover rates in rainbow trout (*Onchorhynchus mykiss*) (Fernández-Navarro et al. 2006).

## Tomato

Tomato (*Solanum lycopersicum* L. or *Lycopersicon esculentum* L.) has achieved tremendous popularity over the last century and is now one of the most important fruit crops. It is cultivated in practically every country in the world—in outdoor fields, glasshouses and nethouses.

### Triterpenoid composition of the tomato skin

**Fig. 4:**
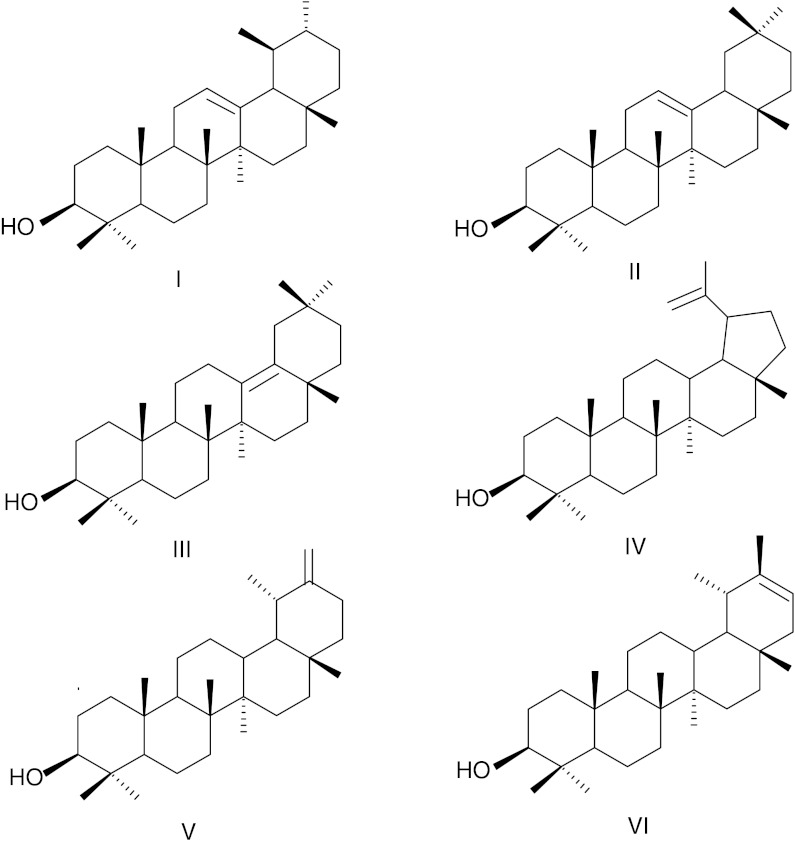
Structures of some triterpenoids occurring in tomato (*L. esculentum*) fruit cuticular waxes: **I** α-amyrin, **II** β-amyrin, **III**, δ-myrin, **IV**, lupeol, **V**, taraksasterol, **VI**, ψ-taraksasterol

In contrast to many other fruits, triterpenoid content of tomato cuticular waxes has been studied in great detail. The occurrence of α-, β- and δ-amyrin (Fig. 4), taraxerol, taraxasterol, ψ-taraxasterol, lupeol, multiflorenol, germanicol, bauerenol, cycloartenol, stigmasterol and β-sitosterol (Table 1) was reported in cuticular wax extracted from the surface of tomato fruits (Bauer et al. 2004a). It is noteworthy that the chemical composition of the triterpenoid fraction of tomato fruit cuticular waxes is strikingly different from the previously described cuticular waxes of apple, grape berries and olive, where triterpene acids (maslinic, oleanolic and ursolic) are the most abundant compounds. These acids do not occur in tomato, where α-, β- and δ-amyrins are the predominant compounds, with quantities that vary considerably in different cultivars and during the subsequent stages of fruit development (Bauer et al. 2004b).

### Studies on the biosynthesis and function of triterpenoids in tomato fruit wax

Diverse cultivars and mutants of tomato have been collected over several decades, and almost one thousand monogenic stocks have been described so far, including mutants of the famous MicroTom, a miniature tomato with a rapid life cycle that is used as a model cultivar for functional genomics (Emmanuel and Levy 2002). Thus, strong genetic and genomic tools are available to investigate processes associated with tomato fruit development and ripening, including the biosynthesis of cuticle lipids and subsequent composition diversity during fruit ontogeny (Mintz-Oron et al. 2008; Kosma et al. 2010; Wang et al. 2011), as well as the correlation between the chemical composition of the cuticular waxes and their function as a transpiration barrier (Vogg et al. 2004; Leide et al. 2007; Leide et al. 2011). In contrast to the vegetative organs, very little molecular information is available regarding the biosynthetic pathways that are active in the surfaces of reproductive organs such as fleshy fruits. Comparative transcriptome and metabolome analyses carried out on peel and flesh tissues during tomato fruit development revealed that different tissues play diverse roles in fruit ripening. Triterpenoids appear to be formed during early fruit expansion rather than full ripening, since the levels of the most abundant triterpenols (α-, β- and δ-amyrins) increased most significantly 25–42 days after flower anthesis (Mintz-Oron et al. 2008). Triterpenoid levels were found to nearly triple from the small green to the mature green stages of tomato fruit development, but changed only slightly during the transformation to the red ripe stage (Kosma et al. 2010). Two genes involved in biosynthesis of triterpenoids have been isolated from tomato fruits and functionally characterized by heterologous expression in yeast as well as overexpression in tomato. One encodes a β-amyrin synthase, which produced β-amyrin as a single product, while the other encodes a multifunctional oxidosqualene cyclase, which yielded a triterpenol mixture containing δ-amyrin (48 %), β-amyrin (13 %), α-amyrin (18 %), multiflorenol (7 %), ψ-taraxasterol (3 %), taraxasterol (6 %) and two unindentified triterpenoids (5 %). The product profile of these two enzymes resembles the range and relative amounts of triterpenoids found in the fruit cuticle. Both enzymes were found to be expressed exclusively in the epidermis of the tomato fruit, indicating that their major function is to form the cuticular triterpenoids. However, the ratios of the specific transcripts did not fully explain the variation in the fruit triterpenoid profiles of different tomato cultivars, with a δ-amyrin: β-amyrin: α-amyrin ratios of 3:3:2 in MicroTom and 3:2:2 in most other cultivars (Wang et al. 2011). Some mutants have a triterpenoid profile that is slightly different from wild-type plants. For example α-, β- and δ-amyrin, β-amyrin derivative, lupeol derivative I, multiflorenol, taraxasterol, ψ-taraxasterol, lanosterol and stigmasterol were identified as common constituents of the fruit waxes of wild-type MicroTom and its *lecer6* mutant (defective in a β-ketoacyl-coenzyme A synthase), whereas β-sitosterol and taraxerol were detected exclusively in the wild-type fruit (Table 1), and the occurrence of lupeol and lupeol derivative II was restricted to the cuticular wax of *lecer6* fruits (Leide et al. 2007).

The *lecer6* mutant was used to study the consequences of a deficiency in very-long-chain aliphatics synthesis for fruit wax composition and the properties of the cuticle as a transpiration barrier. The total amount of wax was only slightly reduced in the mutant plants, but n-alkanes with chain lengths beyond C30 were missing. The decrease in aliphatic components was compensated by a three to six fold increase in the level of triterpenoids, mainly α-, β- and δ-amyrins, accumulated in the intracuticular layer. Experimental removal of the epicuticular wax layer, accounting for one-third of the total wax cover on wild-type fruits, had only a moderate effect on transpiration, whereas the reduction of intracuticular aliphatics in the mutant caused a four fold increase in cuticle permeability. It was concluded that the major portion of the transpiration barrier is located in the intracuticular wax layer and is largely determined by its aliphatic constituents, since an increase in the level of cuticular triterpenoids could not compensate for the loss of aliphatics. Moreover, the composition of the wax mixture also influences the morphological structure of cuticular waxes, which is an important determinant of cuticle permeability. The wax barrier consists of impermeable clusters of crystalline zones embedded in a matrix of amorphous material, and the diffusion of water occurs mainly in the amorphous volume fractions. It was postulated that triterpenoids are localized exclusively in the amorphous zones, so the increase in their content caused spatial rearrangements, and this was the direct reason for the increase in cuticular transpiration (Vogg et al. 2004). The level of triterpenoids was found to differ significantly in wild-type MicroTom and its *lecer6* mutant during the course of fruit maturation. The content of these compounds was initially similar in immature green fruits of both plants (<20 % of total wax), but at the stage of mature green fruit, the level of triterpenoids increased sharply to 80 % of total wax in *lecer6* and it remained at such a high level during all stages of fruit development until the red ripe and red overripe phases. In the wild-type plant, the triterpenoid content peaked in the mature green phase at 53 % of total wax, and then displayed a continuous decline to 21 % in red overripe fruit. With the exception of immature green fruits, the *lecer6* mutant exhibited three to eight fold increased water loss compared with the wild-type, thus confirming a direct relationship between the properties of the cuticular transpiration barrier and chemical modifications in cuticular wax composition (Leide et al. 2007). These findings were later confirmed in a comparison of wild-type tomato culivars John Baer and Pearson with their respective positional sterile (*ps*) mutants, which besides functional male sterility also showed striking phenotypic similarity to the MicroTom *lecer6* mutant. The content of pentacyclic triterpenes and sterol derivatives in wild-type and *ps* mutant fruit cuticular waxes was 19 and 44 %, respectively, with the most prominent triterpenoids being α-, β- and δ-amyrins in wild-types, and β-amyrin and cholesterol in *ps* mutants. The *ps* mutant fruit showed a five to eight fold increase in water permeability compared with the corresponding wild-type tomatoes (Leide et al. 2011).

Recently, wild relatives of cultivated tomato have been studied to evaluate the relationship between evolution, structure and function of the cuticle. These species, *Solanum* Sect. *Lycopersicon*, evolved from a common ancestor about 7 million years ago and today they are endemic to an array of environments in the northern Andes and Galapagos Islands. They can still be crossed with *S. lycopersicum* and share a high degree of genomic synteny. However, striking differences in cuticular architecture and the quantities of cutin and waxes have been observed, with the wax coverage of the wild varieties exceeding that of cultivated tomatoes by up to seven fold. Triterpenoids were found to be the major non-aliphatic compounds of the fruit cuticles, accounting for 1–35 % of the total wax, although they could not be detected in one species, *S. pennellii*. The predominant isomers were α-, β- and δ-amyrins, with ψ–taraxasterol and taraxasterol present at much lower levels. However, in *S. habrochaites*, β-amyrin was the only pentacyclic triterpenoid detected. A correlation between a decreasing prevalence of triterpenoids and phylogenic distance from *S. lycopersicum* was identified. In addition, it was confirmed that besides evolutionary adaptation, environmental growth conditions can have a substantial effect on cuticle properties (Yeats et al. 2012).

The main health benefits correlated with the consumption of tomatoes (prevention of certain types of cancer, liver disorders, heart disease, osteoporosis, cataracts) are usually ascribed to the occurrence of lycopene and β-carotene, vitamins and potassium. However, a number of pharmacological properties, such as antiallergic, antidepressant, antiinflammatory, antinociceptive, antipruritic, anxiolytic, gastroprotective and hepatoprotective activities of the amyrins (monohydroxy triterpene alcohols occurring in a variety of plant materials including tomato cuticular waxes) have been reported (Soldi et al. 2008; Melo et al. 2010; Ching et al. 2011). The mixture of amyrins employed in these studies was usually obtained from leaves or plant resins. If current research on potential pharmacological applications of amyrins produces promising results, it may be economically justified to devise methods to obtain these compounds from by-products, like tomato peel, that remain after the annual processing of millions of tonnes of tomatoes into juice, ketchup, sauce, concentrate and other products.

## Other fruits with edible peel

Edible berries of the genus Vaccinium, including lowbush blueberry *V. angustifolium*, rabbiteye blueberry *V. ashei*, highbush blueberry *V. corymbosum*, cranberry *V. macrocarpon* Ait., bilberry *V. myrtillus* L. and lingonberry (cowberry) *V. vitis*-*idaea* L. are valued for the high content of phenolic antioxidants and numerous health benefits ascribed to their consumption (Moyer et al. 2002; Ono et al. 2004; Neto 2007). Moreover, the occurrence of triterpenoids, mainly ursolic and oleanolic acids and their derivatives, is also well documented for some of these fruits (Murphy et al. 2003; He and Liu 2006; Szakiel and Mroczek 2007; Szakiel et al. 2007; Kondo et al. 2011, Szakiel et al. 2012). Cranberry, native to North America, has attracted public interest as a functional food preventing bacterial adhesion in urinary tract infections and stomach ulcers, protecting against lipoprotein oxidation, reducing cholesterol level and showing in vitro anticancer activity (He and Liu 2006). Together with apple, pear, cherry and prune, cranberry cuticular wax is regarded as one of the richest natural sources of ursolic acid (Beindorff et al. 2001). As early as 1934, the wax extracted from cranberry pomace was shown to contain significant amounts of this compound and thus “crude ursolic acid” started to be produced commercially from a waste product of cranberry juice production. Later, more detailed study also revealed the presence of considerable amounts of oleanolic acid, methyl ursolate and methyl oleanolate, α- and β-amyrins and their acetates, as well as β-sitosterol and stigmasterol, altogether comprising 50 % of a chloroform extract (Table 2). At that time, the highly complex composition of fruit cuticular wax, and especially the occurrence of several classes of triterpenoids with different ratios of ursane and oleanane skeletons in each of them, seemed somewhat unusual (Croteau and Fagerson 1971). Recently, two hydroxycinnamate esters of ursolic acid, occurring in ethyl acetate extracts from whole cranberry fruit and its products containing peel, were shown to possess antiproliferative activities against several types of tumor cells in vitro, including MCF-7 breast, HT-29 colon, DU-145 prostate, H460 lung, ME180 cervical and K562 leukemia cell lines. Cranberry juice and juice-derived sauce appear not to contain significant amounts of these compounds (Kondo et al. 2011; Neto 2011).

**Table 2:** Triterpenoid profile of cuticular waxes of some edible and inedible fruits

Fruit	Triterpenoid profile	Amount	Predominant compounds	Method of wax extraction	Method of triterpenoid identification	References
Asian pear (*P. bretchneideri* Rehd cv. Pingguoli)	α-amyrin; hop-22(29)-en-3β-ol; 14-methyl-ergosta-8,24(28)-dien-3-ol; A′-neogammacer-22(29)-en-3-one; A-neooleana-3(5),12-dien; 14,17-nor-3,21-dioxo-β-amyrin; 6a,14a-methanopicene; oleana-11,13(18)-diene; stigmasta-3,5-diene; urs-12-en-28-al	33.6 % of the total wax extract	α-amyrin (14.2 % of wax extract)	Immersion and agitation of whole fruits in various solvents, i.e. methanol, ether, n-hexan, chloroform, chloroform:methanol (3:1)	GC–MS	Yin et al. (2011)
Bell pepper (*Capsicum annuum* L.)	α-amyrin; β-amyrin; δ-amyrin; bauerenol; campesterol; 3β-friedelanol; friedelin; germanicol; glutinol; isobauerenol; isomultiflorenol; lupeol; multiflorenol; β-sitosterol; stigmasterol; taraxasterol; ψ-taraxasterol; taraxerol	34.9 % of the total wax extract (average from 12 cultivars)	α-amyrin (11.2 % of wax extract, i.e. 33.2 % of total triterpenoids); β-amyrin (9.3 % of wax extract, i.e. 27.7 % of total triterpenoids)	Dipping of whole fruits into *tert*-butylmethyl ether in ultrasonic bath	GC–MS	Bauer et al. (2005)
Cranberry (*V. macrocarpon* Ait.) cv. Howes	α-amyrin; β-amyrin; α-amyrin acetate; β-amyrin acetate; methyl oleanolate; methyl ursolate; oleanolic acid; β-sitosterol; stigmasterol; usolic acid,	47.6 % of the total wax extract	Ursolic acid (20 % of the total wax extract)	Immersion of whole fruits in chloroform	GC–MS	Croteau and Fagerson (1971)
Eggplant (*S. melongena* L.)	α-amyrin; β-amyrin; germanicol; lupeol; β-sitosterol; stigmasterol	2.9 % of the total wax extract (average from 3 cultivars)	Lupeol (0.74 % of wax extract)	Dipping of whole fruits into *tert*-butylmethyl ether in ultrasonic bath	GC–MS	Bauer et al. (2005)
*E. globulus* Labill.	3-acetyl-oleanolic acid; betulinic acid; betulonic acid; 3β,11α-dihydroxyurs-12-en-28-oic acid; 3β-dihydroxyurs-11-en-13β(28)-olide (ursolic acid lactone); oleanolic acid; ursolic acid	34.4 % of the total wax extract	Ursolic acid (17.1 % of wax extract)	Dipping of whole fruits into boiling mixture of acetone/light petroleum (1:1)	GC–MS	Pereira et al. (2005)
Grapefruit (*Citrus paradisi* Macf.) cv. Marsh	α-amyrin; β-amyrin; α-amyrin acetate; β-amyrin acetate; α-amyrone; β-amyrone; friedelin; 24-methylenecycloartanol	49.3 % of the total wax extract	Friedelin (27.9 % of wax extract)	Immersion of whole fruits in chloroform	GC–MS	Norby and McDonald (1994)
Sweet cherry (*P. avium* L.) cv. Kordia	α-amyrin; hederagenin and its isomers*; oleanolic acid; ursolic acid; uvaol	75.6 % of the total wax extract (in mature fruit)	Ursolic acid (60 % of wax extract); oleanolic acid (7.5 % of wax extract)	Extraction of enzymatically isolated cuticle with chloroform	GC–MS	Peschel et al. (2007)

* Tentative identification

*GC*–*MS* gas chromatography–mass spectrometry

Ursolic acid is the predominat compound in the fruit cuticular wax of sweet cherry (*Prunus avium* L.). Studies on the composition of cuticular waxes during sweet cherry fruit development showed that triterpenoids represented the main class of wax constituents (75.6 % of total wax in mature fruit), with ursolic acid accounting for 60 % and oleanolic acid for 7.5 %. Triterpene alcohols of the ursane type (α-amyrin and uvaol) were present in smaller amounts (0.4 and 0.8 %, respectively), and some other triterpenoids could not be identified by GC–MS due to their low abundance (Table 2). The initially very high level of triterpenoids in sweet cherry fruit cuticular wax (92.6 % at 22 days after full bloom), decreased by 18 % at 85 days of fruit development to the level characteristic for the mature stage (Peschel et al. 2007). Thus, *P. avium* displays a pattern of cuticle development that resemble that of many other non-climacteric fruits, which after depositing maximum levels of wax very early in fruit development, exhibit decreasing amounts of wax during ripening.

As shown for tomato, triterpene acids are not always present in fruit cuticular waxes. In Asian pear fruit (*Pyrus bretchneideri* Rehd), triterpenoids constituted 33.6 % of the total wax, with α-amyrin and urs-12-en-28-al being the predominant compounds in a triterpenoid profile comprising 10 constituents (Table 2) (Yin et al. 2011). In bell pepper (*Capsicum anuum* L.), 18 triterpenoid compounds were identified (Table 2), accounting for 34.9 % of the total wax extract, with α-amyrin as the most abundant compound (33.2 % of total triterpenoids) followed by β-amyrin (27.7 %) (Bauer et al. 2005).

## Edible fruits with inedible peel

**Fig. 5:**
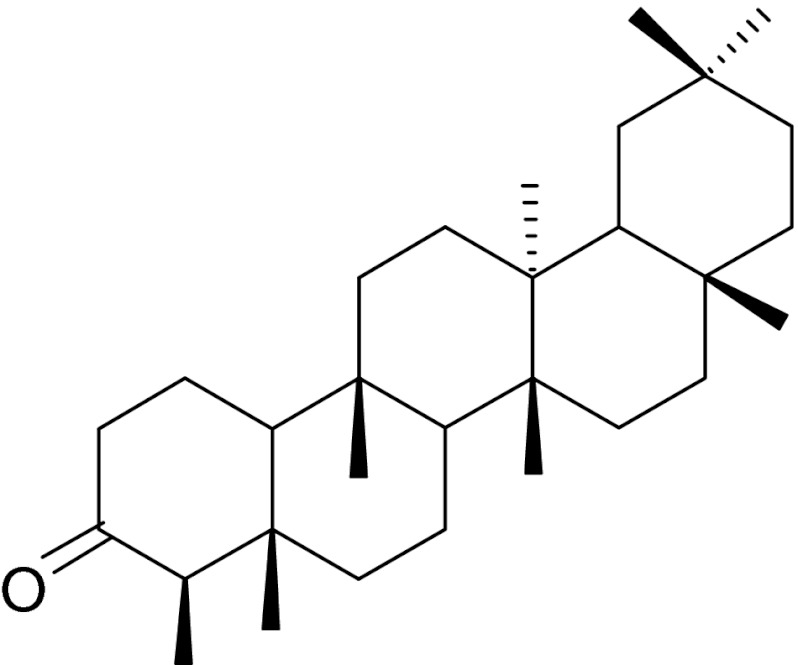
Structure of friedelin occurring in fruit cuticular waxes of grapefruit (*C. paradisi*)

Oranges, grapefruits, mandarines and other fruits of the genus *Citrus* L. are generally peeled when eaten fresh or consumed in the form of juice. Therefore, cuticular waxes of these fruits have been investigated not because of their nutritional value, but in relation to surface disorders like rindstaining, peel pitting or chilling injury, which can cause deterioration of fruit quality. Among cuticular constituents of orange (*C. sinensis* L. Osbeck), tangerine (*C. reticulata* Blanco) and lemon (*C. limon* L. Burm.) harvested in central Florida, triterpenoids were found to constitute a minor fraction, with notable amounts of ursolic acid (Freeman et al. 1979). During fruit ripening, the triterpenoid content of total cuticular wax ranged between 3.3 and 9.4 % in Satsuma mandarins growing in the area of Valencia, and between 3.3–8 % and 3.7–13.6 % in rindstaining-susceptible and non-susceptible Navelina oranges, respectively (Sala et al. 1992). Similarly, triterpenoids were a minor class of fruit cuticular wax component in Fortune mandarins (*C. clementina* × *C. reticulata*) grown in the same region, although their abundance in citrus fruits varied between cultivars and within the same cultivar grown in orchards in different geographical locations. Nevertheless, triterpenoids were never predominant compounds in any orange or mandarin cultivar (Sala 2000). In contrast, triterpenoids were found to be the most prevalent compounds in the fruit cuticular wax of grapefruit (*C. paradisi* Macf.), where they account for 50 % of total wax. Furthermore, ursolic acid was not detected in grapefruit cuticular waxes, and the predominant compound was identified as the triterpene ketone, friedelin (Norby and McDonald 1994). Friedelin (Fig. 5) was reported to be present in grapefruit peel as early as 1955, and is probably the first triterpene ketone to be described in fruit cuticular wax. Recently, the biological properties of this compound, including antiinflammatory, analgestic and antipyretic activities, were investigated (Antonisamy et al. 2011). Other triterpenoids, including α- and β-amyrin, α- and β- amyrin acetate, the ketones: α- and β-amyrone, and 24-methylenecycloartanol (Table 2) were also detected in the fruit cuticular wax of Marsch grapefruit grown in central Florida (Norby and McDonald 1994). Lupeol and two other compounds, tentatively assigned as D:B-friedo-B′:A′-neogammacer-5-en-ol and D:C-friedooleanen-3-one (another ketone), were subsequently added to this triterpenoid profile (Norby and McDonald 1995). Thus, grapefruit peel is characterized by particularly high levels of triterpenones (36 % of total wax).

Studies on fruits belonging to the genus *Citrus* have revealed that the chemical composition of cuticular wax can be very different regardless of systematic relationships. Likewise, the triterpenoid content in fruit cuticular waxes of the eggplant (*Solanum melongena* L.), which accounted for only 2.9 % of the total wax extract, is surprisingly low compared with other members of the family Solanaceae, such as tomato and bell pepper (Bauer et al. 2005).

## Inedible fruits

The tree *Eucalyptus globulus* Labill. is exploited for its wood, which is used by the pulp and paper industry, and for the essential oil extracted from the leaves. After harvesting the wood and leaves, large quantities of unwanted biomass, including the fruits, are discarded. Knowledge of the chemical composition of these unused plant parts could help to solve the problem of this waste material and increase the economic value of *E. globulus* cultivation. The cuticular wax of *E. globulus* fruit was shown to contain a significant amount of triterpenoids (34.4 % of the total wax extract), including ursolic acid, which accounted for 17.1 % of total wax, and an interesting series of other triterpene acids, such as 3-acetyl-oleanolic acid, betulinic acid, betulonic acid, 3β-dihydroxyurs-11-en-13β(28)-olide (ursolic acid lactone), oleanolic acid and 3β,11α-dihydroxyurs-12-en-28-oic acid (Table 2), the last of which was identified for the first time (Pereira et al. 2005). Betulinic acid, usually obtained from the bark of the white birch *Betula alba* L., has been shown to possess a variety of biological properties including antibacterial, anti-HIV, antimalarial, antiinflammatory and anthelmintic activities (Yogeeswari and Sriram 2005). Betulonic acid and its derivatives have been studied as a new group of agents that reduce the side effects of cytostatics (Sorokina et al. 2004; Vasilevsky et al. 2011). Therefore, the cuticular waxes of *E. globulus* fruit contain at least four biologically active triterpene acids (betulinic and betulonic, in addition to oleanolic and ursolic) that may be utilized rather than wasted.

## Concluding remarks

Epidemiological studies have shown that regular consumption of fruit and vegetables is associated with a reduced risk of chronic diseases occurring in the aging human population. The health benefits of plant-based foods are attributed to bioactive phytochemicals, including triterpenoids and other groups of natural compounds, acting in an additive and synergistic way (Liu 2003). No single compound can replace the combination of various phytonutrients, playing their complementary or synergistic roles together. Fruits are a good example of such functional mixtures of water-soluble compounds, including many antioxidants occurring in the flesh, and lipophilic constituents of the cuticle.

Fruit cuticular waxes are receiving growing attention because they can have a significant influence on the production, storage and processing of agricultural commodities. Moreover, compositional studies on these waxes have revealed the occurrence of many biologically active triterpenoids, which, in several cases, accummulate to high concentrations, making the fruit peel an attractive and readily available source of these compounds.

Over the last decade, there has been a increasing interest in pharmaceutical, nutraceutical and cosmeceutical applications of triterpenoids, and they are currently under development as therapeutic agents in numerous treatments, and as functional compounds in many healthcare products. Triterpenoids are highly multifunctional and thus promising in the chemoprevention and chemotherapy of cancer and as antiparasitic agents. However, their activities have usually been demonstrated only in vitro, and although there are extensive preclinical data to support the supposed anticancer properties of triterpenoids, only clinical studies can fully validate them (Yadav et al. 2010). Due to the low polarity and consequently poor aqueous solubility of triterpenoids occurring in free and ester forms, their real bioavailability has been questioned (Jeong et al. 2007; Ganbold et al. 2010; Rada et al. 2011), and formulations based on nanotechnologies have been proposed to replace conventional dosage forms (Chen et al. 2011). At the same time, numerous triterpenoid derivatives with increased bioactivity and improved bioavailability have been synthesized by structural modifications of natural compounds (Bishayee et al. 2011; Sporn et al. 2011).

Since triterpenoids, both in their natural forms and as templates for synthetic modification, are currently of interest to both researchers and industry, natural sources supplying high concentrations of these compounds are in demand. Food industries produce large volumes of wastes, the disposal of which is problematic and may potentially cause environmental pollution. Millions of tonnes of apple, grape berry, olive, tomato, orange and other fruit peels are generated each year as agro-industrial waste which may be utilized to produce various useful materials, fuels and chemicals (Wolfe and Liu 2003; Zhang et al. 2004; Djilas et al. 2009; Ángel Siles López et al. 2010), including triterpenoid-containing products. Fruit pomace is often composted and used as a fertilizer, but sometimes (e.g. grape berry residues remaining after wine-making) it can have an adverse ecological impact due to the allelopathic activities of phenolic compounds inhibiting seed germination (Amico et al. 2004). As the human population grows and natural resources diminish, environmentally valuable and profitable technologies for obtaining natural basic compounds from available waste products are likely to become important for a sustainable future.
